# Characterizing the Emergence of Medetomidine in Southern California Fentanyl Markets

**DOI:** 10.64898/2026.10.13.26365459

**Published:** 2026-10-22

**Authors:** Adam J. Koncsol, Joseph R. Friedman, Caitlin A. Molina, Megan Grabill, Morgan E. Godvin, Ruby Romero, Elizabeth A. Samuels, Edward Sisco, Chelsea L. Shover

## Abstract

**Background:**

Medetomidine, a potent sedative, is increasingly present in fentanyl-related hospitalizations and deaths. Multiple states have passed legislation to control xylazine—a pharmacologically similar sedative in the illicit opioid supply. Medetomidine may function as a highly potent substitute for xylazine with significant overdose risk and withdrawal potential. Medetomidine has previously been described mostly in eastern U.S. fentanyl markets. Here, we characterize medetomidine-containing drug samples obtained in Southern California 2023–2026.

**Methods:**

Anonymous participants in Southern California voluntarily provided drug products to a community-based drug checking program. All samples underwent direct-analysis-in-real-time mass spectrometry (DART-MS). A subset was quantified by liquid-chromatography tandem mass spectrometry (LC-MS/MS). A smaller subset underwent enantiomeric analysis. We calculated other compounds co-present with medetomidine, the prevalence of medetomidine in fentanyl over time, the concentration of medetomidine, and the enantiomer makeup of medetomidine.

**Results:**

The quarterly prevalence of medetomidine co-present with fentanyl rose from ≈6 % in May 2025 (month of first detection) to ≈25 % by August 2026. The average medetomidine concentration was 0.4 % mass fraction. The most common co-detected substances with medetomidine were fentanyl and adulterants such as lidocaine and xylazine. All medetomidine samples that underwent enantiomeric analysis (n = 13) were identified as racemic.

**Conclusion:**

Medetomidine represents a rapidly emerging adulterant in the Southern California fentanyl supply. All samples assessed for enantiomer status were racemic medetomidine, suggesting they were not diverted from human pharmaceutical or medical supplies. Given its high potency, a small amount (<1.5%) is likely still a physiologically significant quantity of the drug.

**Highlights:**

Medetomidine is an emerging adulterant in the Southern California fentanyl supply.
The share of fentanyl with medetomidine rose from 6% in May 2025 to 25% in Aug 2026.
Nearly all medetomidine samples were detected in the presence of fentanyl.
Medetomidine was found in two samples of DMT obtained via the dark web.
The mean concentration of medetomidine was 0.4 % mass fraction among 22 cases.
All tested medetomidine was racemic, not diverted dexmedetomidine.

